# Validating Discriminative Signatures for Obstructive Sleep Apnea in Exhaled Breath

**DOI:** 10.3390/cells11192982

**Published:** 2022-09-24

**Authors:** Bettina Streckenbach, Martin Osswald, Stefan Malesevic, Renato Zenobi, Malcolm Kohler

**Affiliations:** 1 ETH Zurich, Department of Chemistry and Applied Biosciences, 8093 Zurich, Switzerland; 2 Department of Pulmonology, University Hospital Zürich, 8091 Zurich, Switzerland

**Keywords:** breath analysis, biomarkers, mass spectrometry, obstructive sleep apnea, SESI-HRMS, validation

## Abstract

Rapid and reliable tools for the diagnosis and monitoring of obstructive sleep apnea (OSA) are currently lacking. Prior studies using a chemical analysis of exhaled breath have suggested the existence of an OSA-specific metabolic signature. Here, we validated this diagnostic approach and the proposed marker compounds, as well as their potential to reliably diagnose OSA. In this cross-sectional observational study, exhaled breath was analyzed using secondary electrospray ionization high-resolution mass spectrometry. The study cohort included untreated OSA patients, OSA patients treated with continuous positive airway pressure and healthy subjects. The robustness of previously reported OSA markers was validated based on detectability, significant differences between groups (Mann–Whitney U test) and classification performance. The breath analysis of 118 participants resulted in 42 previously reported markers that could be confirmed in this independent validation cohort. Nine markers were significantly increased in untreated OSA compared to treated OSA, with a subset of them being consistent with a previous validation study. An OSA prediction based on the confirmed OSA signature performed with an AUC of 0.80 (accuracy 77%, sensitivity 73% and specificity 80%). As several breath markers were clearly found to be repeatable and robust in this independent validation study, these results underscore the clinical potential of breath analysis for OSA diagnostics and monitoring.

## 1. Introduction

Obstructive sleep apnea (OSA) is a sleep-related breathing disorder with high prevalence at an advanced age [1]. During sleep, repetitive complete or partial collapses of the pharynx result in short events of apneas or hypopneas, respectively. These apnea/hypopnea events are associated with oxygen desaturations and arousals from sleep that impair the quality of sleep and, thus, may cause daytime sleepiness. Symptomatic OSA has been shown to be associated with the development of depression and an increased risk for car accidents [2,3]. Moreover, patients with OSA are at higher risk for developing diabetes, arterial hypertension and cardiovascular diseases [4,5,6]. One of the most efficient treatments of OSA is the nocturnal use of a mask with continuous positive airway pressure to overcome pharyngeal airway obstruction (CPAP) [7], although it is not equally well-tolerated by every patient [8,9].

The gold standard of OSA diagnosis is in-laboratory polysomnography (PSG). Among other variables, the apnea/hypopnea index (AHI) and oxygen desaturation index (ODI) are derived from PSG and form the basis for the clinical diagnosis and, furthermore provide information about the severity of the disease [10]. However, in-laboratory sleep studies are time consuming, costly and bothersome for the patient, which may affect the patient’s sleep and, thus, the sleep study results. Questionnaires such as the Berlin questionnaire, STOP-Bang or the NoSAS score have been used for the screening of OSA [11,12,13]. However, most questionnaires are subjective and allow for screening only, as final diagnosis still relies on sleep studies. Furthermore, they are not intended to be used for monitoring the treatment success. For this purpose, the Epworth Sleepiness Scale (ESS) questionnaire is routinely applied [14]. However, this questionnaire is entirely subjective and has been shown to inherit day-to-day as well as within-day variations [15]. 

For OSA, current research on clinical tools focuses on simplifying objective screening in primary care and improving the diagnosis of OSA without the need for time-consuming in-laboratory sleep studies. In this regard, the NoSAS score was introduced as a simpler screening tool using easily accessible clinical variables and, in different studies, showed similar or superior performances compared with previous OSA screening methods [13,16,17]. This five-item questionnaire includes snoring, which requires the statement of a bed partner. The same trend was followed by the four-item tool GOAL and the two-item tool No-Apnea, but their performance lacks validation in multi-ethnic populations [18,19]. The home sleep apnea test (HSAT) represents an alternative to PSG for OSA diagnosis but was recommended only for a subset of patients [20]. Various candidate biomarkers for OSA have been reported and linked to metabolic symptoms of OSA such as diabetes mellitus, oxidative stress or inflammation, but reliable biomarkers are not available at this stage [21]. 

Exhaled breath is known to contain hundreds of volatile organic compounds (VOCs) that can be detected with modern analytical methods [22]. Some of these VOCs are metabolites of endogenous origin and can give metabolic insights into the human physiological state. Together with the non-invasiveness of breath sampling, breath analysis has therefore gained increasing interest for clinical applications. One of the few examples where breath analysis is used in the clinical environment is the quantification of fractional exhaled nitric oxide (FeNO) as a biomarker for airway inflammation [23]. However, disease-specific biomarkers in breath are not yet routinely being used in the clinics. 

Two prior studies that employed online breath analysis showed promising results for OSA diagnostics. In a randomized controlled trial, secondary electrospray ionization high-resolution mass spectrometry (SESI-HRMS) revealed a metabolic pattern in exhaled breath that was characteristic for OSA recurrence upon CPAP withdrawal [24]. Moreover, some of these molecules correlated well with the AHI or ODI values. Parts of this breath pattern were successfully validated in a more recent observational study including a larger and more heterogenous study cohort of subjects with and without OSA [25]. 

For the successful introduction of SESI-HRMS as a clinical screening and diagnostic tool as well as for monitoring of metabolic treatment effects, further validation of the previously identified breath markers is needed. Therefore, the present cross-sectional observational study aims at validating the previously reported OSA breath biomarkers in another independent cohort consisting of treated and untreated OSA patients as well as control subjects without OSA.

## 2. Materials and Methods

### 2.1. Study Participants and Clinical Data

Patients from the University Hospital Zurich and the Verein Lunge Zürich database were screened for eligibility in this cross-sectional study. All patients with respiratory polygraphy or polysomnography reports from the sleep laboratory were considered for one of the three study groups: (i) patients diagnosed with OSA but without treatment, (ii) patients diagnosed with OSA and have been receiving CPAP treatment for at least three months, and (iii) patients with no OSA as defined by polysomnography or respiratory polygraphy. Central and mixed sleep apnea, as well as further comorbidities and conditions were excluded, as listed in Table S1. Measurements of all three study groups were performed within the same period. At the day of breath analysis, demographic data and information on lifestyle, medications and comorbidities were collected. In addition, the ESS questionnaire (German version) [26] was completed by the participants. Clinical data at diagnosis were retrieved from the sleep laboratory report of each participant, which included reports for the treated OSA group before onset of the CPAP treatment. Subjects of the untreated OSA group were supplied with an apnea link to retrieve recent AHI values. For subjects of the treated OSA group, their CPAP usage was obtained by the CPAP-machine chip readout and averaged over the past three months. The study was performed in accordance with the Declaration of Helsinki, and all subjects gave written informed consent before participation in their first visit. The study was registered at ClinicalTrials.gov (NCT05456009) and approved by the local ethics committee (KEK-ZH 2019-00030).

### 2.2. Direct Breath Analysis

Exhaled breath was analyzed using a SuperSESI ion source (FossilionTech, Madrid, Spain) coupled to a high-resolution mass spectrometer (TripleTOF 5600+, AB Sciex, Concord, ON, Canada). All subjects were requested to refrain from eating, drinking (except water) and brushing their teeth for at least 1 h prior to the measurements. For breath measurements, the subjects exhaled directly into the instrument at a constant pressure drop of 10–14 mbar and using a single-use mouthpiece. Each participant’s measurement consisted of six consecutive exhalations. The ion source was heated (sampling line 100 °C, ion core 130 °C), and the net flow through the ion source was set to 0.3 L/min. Mass spectra were recorded in positive ionization mode (4.5 kV) in the mass range of 50–500 Da and with an accumulation time of 0.5 s. Instrument operators adhered to internal standard operating procedures to ensure data comparability throughout the study visit.

### 2.3. Data Preprocessing

Mass spectral data from breath measurements were converted into mzXML files using MSConvert (ProteoWizard v3.0.2) [27]. Preprocessing was then performed in Matlab (version R2022a, MathWorks, Natick, MA, USA) and adapted to a previously described procedure [24]. In brief, spectra of all subjects were re-calibrated on one breath spectrum, and signal intensities were interpolated and aligned. Scans during exhalation were selected based on the presence of the water cluster ion at *m*/*z* = 55.04 ([(H_2_O)_3_+H]^+^), and peak picking was performed on these breath scans with a height filter of 5 counts per seconds. Breath scan intensities of each subject were averaged, normalized to the total ion current (TIC), scaled to the median TIC of all included subjects and auto-scaled, resulting in the final intensity matrix.

### 2.4. Satistical Analysis and Classification

Marker detection analysis was performed on the averaged intensity matrix of all participants with a mass tolerance of 0.005 Da. Only *m*/*z* features reported by Schwarz et al. [24] and validated by Nowak et al. [25], in total 78 features, were considered. Markers with significant differences between subjects of the untreated OSA and treated OSA group were assessed (stratification 1: minimal 5 h/night CPAP usage). Significance testing was performed with the Mann-Whitney U test because the marker intensities were not normally distributed (Shapiro-Wilk test, Figure S1) in the tested study groups. The predictive power of breath features was assessed in a 10-fold cross validation using the Matlab classification learner app and based on all detected *m*/*z* features. The best performing classification algorithm was evaluated pairwise for the untreated OSA and treated OSA group (stratification 2: AHI > 30, or AHI > 10 and ESS > 10, and additionally for treated OSA a minimum of 5 h/night CPAP usage) and for the untreated OSA and control group.

## 3. Results

### 3.1. Study Participants

**Figure 1:**
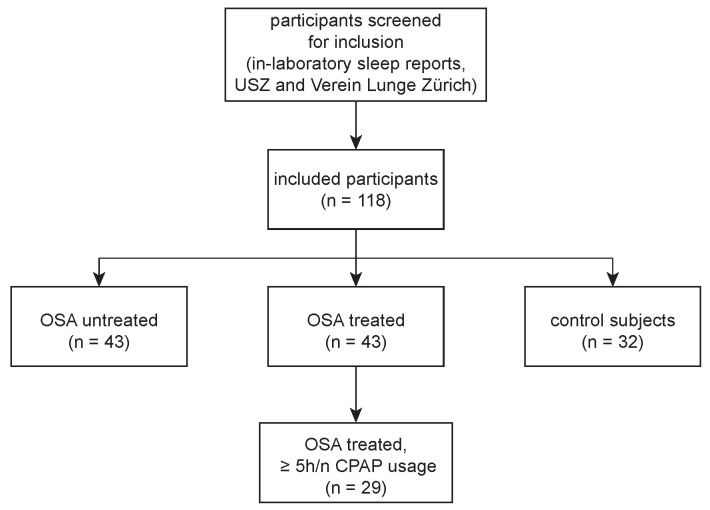
Study profile. In-laboratory sleep reports: polysomnography or respiratory polygraphy. USZ: University Hospital Zurich, OSA: obstructive sleep apnea, OSA treated: patients with OSA and continuous positive airway pressure (CPAP) therapy.

In this study, 118 participants were enrolled, including 43 patients with untreated OSA, 43 patients with treated OSA and 32 control subjects (Figure 1). The demographic data of both the untreated and treated OSA groups were comparable, while the control group without OSA differed in size, age and sex (Table 1). Nevertheless, the body mass indices (BMI) were comparable throughout all groups. The most prevalent comorbidities and concomitant medications are listed in Table S2. The AHI values at the time of study inclusion were significantly higher in the untreated OSA group (28.0 [18.8, 40.0]) compared to both the treated OSA (1.3 [0.8, 3.6]) and control group (3.0 [2.0, 4.0]). Furthermore, the AHI values from the original diagnostic reports (before initiation of CPAP treatment) showed higher AHI and ESS values in the treated compared to the untreated OSA group (Table 1, at diagnosis).

### 3.2. Validation of Breath Signatures

**Figure 2:**
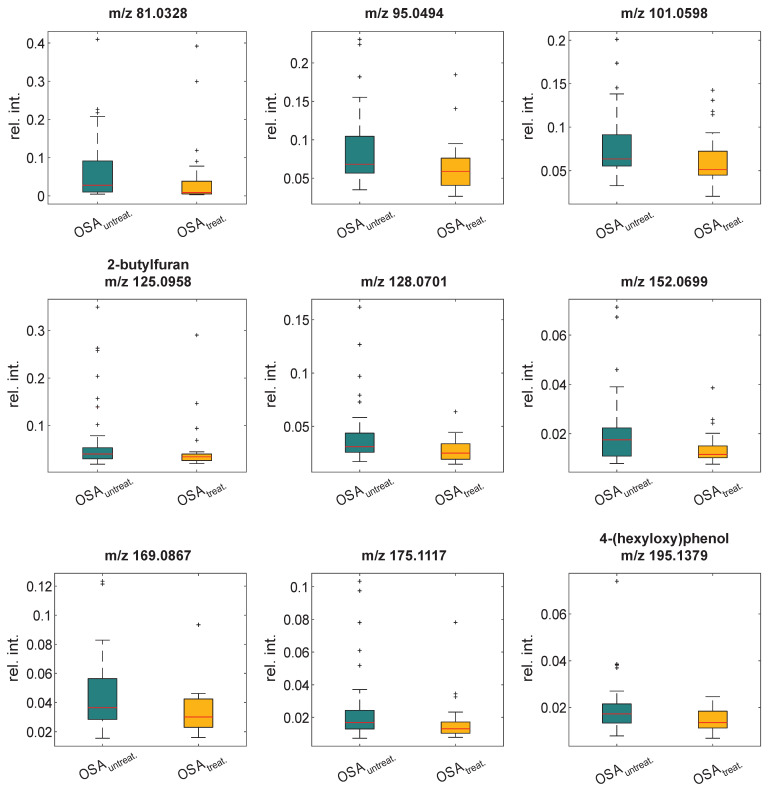
Significantly different markers between the untreated and treated OSA group. Stratification for the treated OSA group (OSA_treat._): averaged CPAP usage ≥ 5 h/night, statistical significance level: *p* < 0.05. Boxplots include the median: red line, 25th and 75th percentiles: bottom and top box edges, 1.5-fold IQR: whiskers, and outliers: +.

Breath analysis by SESI-HRMS of all participants revealed a set of 42 out of 78 previously validated OSA features, which could be confirmed in the current validation cohort (Table S3). Here, *m*/*z* features that had been reported as OSA-associated markers in two independent study cohorts were targeted [24,25]. A difference analysis between the groups was performed on the normalized signal intensities of these 42 metabolites. To ensure that possible metabolic effects attributed to CPAP treatment could be detected, a stratification criterion of a minimum averaged CPAP usage of 5 h/night during the last three months was applied to the treated OSA group. In total, 43 patients with untreated OSA and 29 patients with treated OSA were included, and the characteristics of the groups are summarized in Table S4. The Mann–Whitney U test was applied on the 42 detected features because the data were not normally distributed (Shapiro–Wilk test Figure S1). This resulted in a set of nine markers with significant differences (*p* < 0.05) in their intensities between the two groups (Figure 2 and Figure S2). The present study did not focus on chemical identification of the markers, such that only a couple of them have a chemical name associated with them; most are reported as accurate *m*/*z* values (Table 2). All these markers were increased in the untreated OSA group with four of them confirming the previously reported increase in OSA patients (Table 2).

### 3.3. Classification Based on Breath Signatures

**Figure 3:**
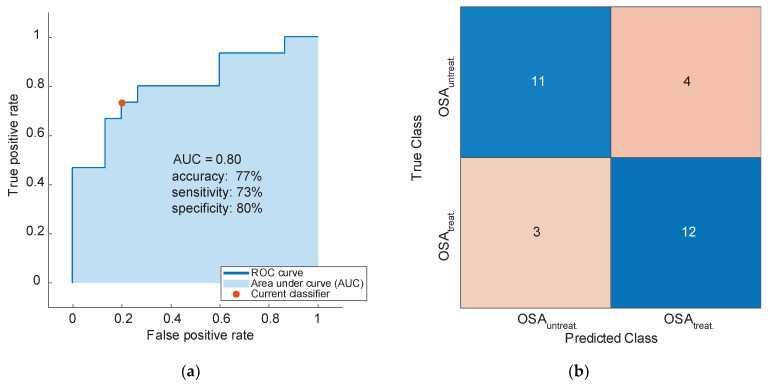
Classification performance of the 42 detected features for untreated OSA (OSA_untreat._) and treated OSA (OSA_treat._) with balanced group size (each n = 15). Stratification criteria: AHI > 30, or AHI > 10 and ESS > 10, additionally for the treated OSA group: averaged CPAP usage ≥ 5 h/night. (**a**) Receiver operating characteristic curve for OSA prediction from a 10-fold cross-validation applying the support vector machine algorithm resulted in an averaged area under the curve (AUC) of 0.80; (**b**) confusion matrix for the prediction of untreated and treated OSA.

The set of markers we confirmed in this study was further evaluated based on their classification performance to predict OSA within the study cohort. The intensities of all 42 markers were included as predictors, and similar stratification criteria as in the previous validation study were applied (AHI > 30, or AHI > 10 and ESS > 10, and additionally for treated OSA: ≥5 h/night CPAP usage). In a 10-fold cross-validation, the receiver operating curve for OSA prediction yielded an area under the curve (AUC) of 0.65, with a prediction accuracy of 64%, sensitivity of 75% and specificity of 53% (Figure S3). Reducing the untreated OSA group randomly for a balanced group size to build the prediction model resulted in an AUC of 0.80 and strongly improved accuracy (77%) and specificity (80%), but at the slight expense of sensitivity (73%) (Figure 3).

Furthermore, we assessed the predictive power of the marker set for screening purposes to discriminate between patients with untreated OSA and control subjects. The model was trained on the marker intensities of patients with untreated OSA (n = 24) and healthy controls (n = 32) and resulted in an AUC of 0.60, with 60% accuracy, 58% sensitivity and 63% specificity (Figure S4). In this case, a balanced group size did not show an improved classification performance (Table S5). 

## 4. Discussion

Real-time breath analysis has been studied increasingly in recent years in view of its potential for clinical application. For instance, the SESI-HRMS technology has been successfully applied in a number of explorative studies on various health conditions [22]. However, validation studies to assess repeatability and robustness of reported outcomes, which are pivotal for the translation into clinical applications, are scarce to date [28]. To the best of our knowledge, this is the first time a second independent study has been conducted to validate results from clinical trials using a SESI-HRMS-based breath analysis.

To this end, we evaluated a set of breath-borne markers associated with OSA, which were discovered while investigating metabolic changes in breath upon OSA recurrence induced by CPAP withdrawal [24] and subsequently validated in a second, observational study (followingly referred to as first validation study) [25]. Initially, more than two hundred *m*/*z* features potentially associated with OSA were reported, out of which 78 molecules were detected in the first validation study. Forty-two of these were detected in this second validation study cohort, with nine of them significantly increased in patients with untreated OSA. This is nevertheless remarkable considering that the instrumentation has not yet been standardized. Several instrument updates were made, including the installation of different and improved SESI sources as well as acquisition settings in each of the three studies on OSA, as previously discussed by Nowak et al. [25]. Specifically, prior to this study, the SESI-HRMS setup was relocated from a research environment at ETH Zurich to the clinical environment of the University Hospital Zurich. As a result, we expected different sensitivities in marker detection and changes in the chemical backgrounds, in addition to the different and independent study cohorts. Thus, the fact that numerous identical *m*/*z* markers characteristic for OSA were found indicates that these markers are robust and relevant. Moreover, four validated markers that were elevated in patients with untreated OSA matched those from the first validation study (Table 2).

Among the nine markers found to be significantly increased in untreated OSA, 2-butylfuran and 4-(hexyloxy) phenol had been identified. Interestingly, two additional furans, 2-ethylfuran and 2-propylfuran, as well as 4-hydroxy-2-octenal were close to the significance level (Table S3). Both derivatives of furans and aldehydes had been unambiguously identified in exhaled breath [29,30]. Here, they showed an increase in patients with untreated OSA, which was, specific to both furans, comparable to that of 2-butylfuran (1.44 FC). Furthermore, 2-butylfuran was consistently elevated in both stratification analyses in the first validation study (Table 2), and in the randomized clinical trial upon CPAP withdrawal and consequent OSA recurrence. This suggests an association of furans observed in the metabolic signature of OSA. Furans in humans have been discussed to originate from gut microbiota, while the constellation of gut microbiota was altered in OSA mice models [31,32]. This would be consistent with increasing evidence for the presence of microbial metabolites in exhaled breath [33]. Furan derivatives in exhaled breath have also been linked to smoking [34,35], which could not be confirmed as the sole origin in the current study cohort because both groups included an equal number of active smokers.

With 4-hydroxy-2-octenal, a representative of aldehydes was detected and showed close to moderate evidence (*p* 0.054) for a significant difference between the treated and untreated OSA groups. Interestingly, aldehydes have been linked to lipid peroxidation induced by oxidative stress [36] and were consistently found to be associated with symptomatic OSA in all three breath analysis studies. Furthermore, they have been identified in several studies in exhaled breath of patients with chronic obstructive pulmonary disease and asthma, reportedly showing discriminative potential for both airway diseases [37].

The exhaled markers for OSA classification exhibited a more specific than sensitive test performance in both cases: predicting untreated OSA against CPAP-treated OSA (80% specificity) as well as predicting untreated OSA against control subjects (63% specificity). Despite similar group sizes, these results are distinct from the first validation study in a higher sensitivity. Therefore, a positive test result is more likely to rule in the disease. However, the fact that we obtained a fairly high false negative rate (FNR) of 42% when comparing untreated OSA to healthy controls suggests that the test is less suitable for initial screening of OSA. Nonetheless, comparing untreated OSA with treated OSA resulted in a lower FNR of 27%, indicating that the test is less likely to miss untreated OSA. Together with the higher specificity, a breath analysis of these OSA-associated markers could be useful as a fast tool to monitor the disease.

There are some considerations to take into account when interpreting the study results. Compared to both previous studies, the classification performance in the current study might have been affected by the reduced number of applicable predictors and a more diverse collection of clinical data: while the ESS questionnaires had been filled in on the same day as the breath measurements, the AHI values were collected at different timepoints and by different tools, i.e., for the control group, from the last sleep study reports, and for the treated OSA group (CPAP-machine readout) and the untreated OSA group (apnea link readout), on the day of the visit. This impaired correlation analysis between detected metabolites and OSA severity and, thus, could not be validated in this study.

While the controlled addition of internal standards has yet to be implemented in SESI-HRMS workflows, technical fluctuations resulting from time drifts were minimized in this study by relocating the instrumental setup to the hospital. This allowed us to reduce the study period for breath analysis of 118 participants to only six weeks.

The OSA-associated markers had been identified in a well-controlled but small study cohort, as common for pilot studies, using prediction modeling among other methods. Machine learning tools for prediction models may allow us to build robust training models despite small sample sizes, but they bear the risk of overfitting [38]. Thus, increased study cohorts would strengthen the selection of predictors and might reveal even more reliable predictors. It follows that compound identification of these predictors will allow for a better understanding of metabolic changes that result in the diverse symptoms of OSA and adds relevance to these OSA-associated molecules from a pathophysiological point of view. Quantifying validated metabolites will be needed to define threshold levels of breath-borne biomarkers for symptomatic OSA. As a result, this would allow us to use breath analysis as a fast tool for primary screening of possible OSA patients and for monitoring of the disease in clinical routines. 

## 5. Conclusions

In this second validation study, we were able to confirm a set of OSA-associated features using SESI-HRMS in exhaled breath. The ability to confirm several previously reported markers despite a diversified study cohort and changes on the instrumental setup and environment strongly advocates the robustness and relevance of these markers in symptomatic OSA. 

## Figures and Tables

**Table 1:** Baseline characteristics.

	OSA Untreated	OSA Treated	Control Subjects	*p* Value ^1^
N	43	43	32	-
Age (y)	61 (55, 69)	61 (53, 69)	50 (39, 56)	0.0002
Sex, male, N (%)	36 (84%)	36 (84%)	19 (59%)	0.020
BMI (kg/m^2^)	29.5 (27.0, 34.0)	30.1 (26.9, 32.3)	29.7 [27.4, 32.4)	0.86
Smoker, N (%)	28 (65%)	29 (67%)	18 (56%)	0.59
Smoking, py	0.0 (0.0, 25.0)	1.0 (0.0, 18.0)	0.0 (0.0, 9.3)	0.85
AHI at diagnosis (events/h)	30.2 (24.0, 45.0)	35.5 (24.0, 44.5)	3.0 (2.0, 4.0)	0.0001
ODI at diagnosis (events/h)	32.0 (23.0, 48.2)	29.0 (16.0, 46.0)	4.5 (2.5, 8.5)	0.0001
AHI at visit (events/h)	28.0 (18.8, 40.0)	1.3 (0.8, 3.6)	n.a.	0.0001
ODI at visit (events/h)	29.9 (13.9, 41.4)	n.a.	n.a.	-
ESS at visit, points	5.0 (3.0, 9.0)	6.0 (3.0, 9.0)	7.5 (6.0, 11.0)	0.055

^1^*p* values were determined for categorical variables using the Chi-square test, for continuous variables using the Kruskal-Wallis test. BMI: body mass index, py: packages per year, AHI: apnea/hypopnea index, ODI: oxygen desaturation index, ESS: Epworth Sleepiness Scale. Values are presented as median ± interquartile range (IQR), unless otherwise stated.

**Table 2:** Significant OSA-associated markers and their detection reported in the previous validation study [25].

Significant Markers				Marker Detection in Previous Study [25]			
				Stratification 1 ^a^		Stratification 2 ^b^	
*m*/*z*	Elemental Composition ^c^	Metabolite	Behavior	Sign.	Behavior	Sign.	Behavior
81.0328	C5H5O	n.a.	increased	no	n.a.	no	n.a.
95.0494	C6H7O	n.a.	increased	no	n.a.	no	n.a.
101.0598	C5H9O2	n.a.	increased	no	n.a.	no	n.a.
125.0958	C8H13O	2-butylfuran	increased	yes	increased	yes	increased
128.0701	C6H10NO2	n.a.	increased	yes	increased	no	n.a.
152.0699	C8H10NO2	n.a.	increased	no	n.a.	no	n.a.
169.0867	C9H13O3	n.a.	increased	no	n.a.	no	n.a.
175.1117	C12H15O	n.a.	increased	yes	increased	yes	increased
195.1379	C12H19O2	4-(hexyloxy)phenol	increased	no	n.a.	yes	increased

^a^ stratification 1: ODI > 30, or ODI > 10 and ESS >10, ^b^ stratification 2: ODI > 30 and ESS > 10, sign.: statistically significant (*p* < 0.05), ^c^ composition suggested based on exact mass except for 2-butylfuran and 4-(hexyloxy)phenol.

## Data Availability

Data are available upon request to the corresponding author.

## Supplementary Materials

The following supporting information can be downloaded at: https://www.mdpi.com/article/10.3390/cells11192982/s1, Figure S1: Shapiro–Wilk test for normality on the detected marker intensities in untreated and treated OSA; Figure S2: Mann–Whitney U test to determine significantly different OSA-associated metabolites between untreated and treated OSA patients; Figure S3: Classification performance of the 42 detected features for untreated OSA and treated OSA; Figure S4: Classification performance of the 42 detected features for untreated OSA and control subjects; Table S1: Inclusion and exclusion criteria for study participants; Table S2: Most prominent comorbidities and concomitant medications. Table S3: Confirmed *m*/*z* features in the validation study; Table S4: Cohort characteristics with stratification criterion applied for the treated OSA group; Table S5: Classification performance of 42 confirmed markers applied on the different study groups.
