# Supplementary material for: Validating Discriminative Signatures for Obstructive Sleep Apnea in Exhaled Breath
Source: Cells. 2022 Sep 24;11(19):2982. doi: 10.3390/cells11192982 (PMC9563926; doi:10.3390/cells11192982)
Supplement: Supplementary file 1 [file cells-11-02982-s001.zip › cells-1904860-supplementary.pdf]

Supplemental Material

# Validating Discriminative Signatures for Obstructive Sleep Apnea in Exhaled Breath

Bettina Streckenbach[1†], Martin Osswald[2†], Stefan Malesevic[2], Renato Zenobi[1], Malcolm Kohler[2*]

[1]ETH Zurich, Department of Chemistry and Applied Biosciences, 8093 Zurich, Switzerland
[2]Department of Pulmonology, University Hospital Zurich, 8091 Zurich, Switzerland

[†] These authors contributed equally to this work.

[*]corresponding author: malcolm.kohler@usz.ch

The following inclusion and exclusion criteria were applied for participant screening as listed in Table S1.

**Table S1:** Inclusion and exclusion criteria for study participants.

<table>
<tr><th></th><th colspan="3">Inclusion Criteria</th><th>Exclusion Criteria</th></tr>
<tr><th>study cohort</th><th>Age</th><th>AHI</th><th>current condition</th><th>condition/ comorbidities</th></tr>
<tr><td>OSA untreated</td><td>≥ 18</td><td>≥ 15[a]</td><td>OSA without CPAP treatment or other treatments for OSA</td><td rowspan="3">central or mixed sleep apnea<br>pregnancy<br>malignom<br>lung cancer<br>lung transplant<br>interstitial lung disease<br>chronic obstructive lung disease (COPD, asthma)<br>hypoventilation (e.g., adipositas hypoventilation)<br>chronic inflammatory disease<br>relevant congenital defect<br>renal failure<br>drug abuse</td></tr>
<tr><td>OSA treated</td><td>≥ 18</td><td>≥ 15[a]</td><td>OSA with CPAP treatment</td></tr>
<tr><td>healthy controls</td><td>≥ 18</td><td>≤ 5</td><td>no clinical OSA</td></tr>
</table>

AHI: apnea/hypopnea index (events/h), OSA: obstructive sleep apnea, CPAP: continuous positive airway pressure, COPD: chronic obstructive pulmonary disease, [a]AHI values at diagnosis.

**Table S2:** Most prevalent comorbidities and concomitant medications.

| | **OSA untreated (N=43)** | **OSA treated (N=43)** | **Control subjects (N=32)** |
|---|---|---|---|
| **Comorbidities** | | | |
| Depression | 6 (14) | 4 (9) | 6 (19) |
| Diabetes mellitus | 8 (19) | 6 (14) | 2 (6) |
| Hypertension | 19 (44) | 23 (53) | 10 (31) |
| Thyroid dysfunction | 1 (2) | 3 (7) | 3 (9) |
| **Concomitant medication** | | | |
| Antidepressants | 7 (16) | 8 (19) | 9 (28) |
| Antidiabetics | 9 (21) | 7 (16) | 4 (13) |
| Antihypertensiva | 27 (63) | 29 (67) | 13 (41) |

Values are presented as number (%) per study group, OSA = obstructive sleep apnea.

# Confirmed OSA-associated metabolites

**Table S3:** Confirmed m/z features in the validation study.

| | m/z | metabolite | Signif. | in untreated OSA | *P* value | FC |
|---|---|---|---|---|---|---|
| 1 | 69.0693 | n.a. | no | | 0.854 | 1.02 |
| 2 | 71.0487 | n.a. | no | | 0.739 | 1.31 |
| 3 | 79.0409 | n.a. | no | | 1.000 | 1.19 |
| 4 | 81.0328 | n.a. | yes | increased | 0.012 | 1.49 |
| 5 | 81.0525 | n.a. | no | | 0.818 | 1.23 |
| 6 | 83.0854 | n.a. | no | | 0.688 | 1.32 |
| 7 | 87.0439 | n.a. | no | | 0.083 | 1.22 |
| 8 | 91.0413 | n.a. | no | | 0.973 | 1.01 |
| 9 | 93.0574 | n.a. | no | | 0.484 | 1.53 |
| 10 | 95.0494 | n.a. | yes | increased | 0.022 | 1.33 |
| 11 | 97.0647 | 2-ethylfuran | no | | 0.079 | 1.44 |
| 12 | 101.0598 | n.a. | yes | increased | 0.031 | 1.22 |
| 13 | 102.0913 | 2-pentenal ([M+NH4]+) | no | | 0.845 | 1.03 |
| 14 | 105.0551 | n.a. | no | | 0.211 | 1.14 |
| 15 | 109.0648 | n.a. | no | | 0.558 | 0.81 |
| 16 | 111.0803 | 2-propylfuran | no | | 0.087 | 1.49 |
| 17 | 123.1165 | n.a. | no | | 0.801 | 1.13 |
| 18 | 125.0958 | 2-butylfuran | yes | increased | 0.029 | 1.44 |
| 19 | 128.0701 | n.a. | yes | increased | 0.006 | 1.49 |
| 20 | 129.0908 | 4-hydroxy-2-heptenal | no | | 0.323 | 1.59 |
| 21 | 136.0216 | benzothiazole | no | | 0.081 | 1.37 |
| 22 | 137.0593 | n.a. | no | | 0.081 | 1.77 |
| 23 | 138.0571 | n.a. | no | | 0.081 | 0.96 |
| 24 | 139.1116 | 2-pentylfuran | no | | 0.491 | 1.15 |
| 25 | 143.1063 | 4-hydroxy-2-octenal ([M+NH4]+) | no | | 0.139 | 3.25 |
| 26 | 149.0971 | n.a. | no | | 0.927 | 1.37 |
| 27 | 151.1116 | n.a. | no | | 0.696 | 0.77 |
| 28 | 152.0699 | n.a. | yes | increased | 0.018 | 1.48 |
| 29 | 160.0611 | n.a. | no | | 0.073 | 1.25 |
| 30 | 160.1329 | 4-hydroxy-2-octenal | no | | 0.054 | 6.13 |
| 31 | 167.1064 | n.a. | no | | 0.513 | 0.92 |
| 32 | 169.0867 | n.a. | yes | increased | 0.020 | 1.36 |
| 33 | 175.1117 | n.a. | yes | increased | 0.037 | 1.42 |
| 34 | 195.1379 | 4-(hexyloxy)phenol | yes | increased | 0.042 | 1.32 |
| 35 | 208.1776 | n.a. | no | | 0.075 | 2.46 |
| 36 | 209.1168 | n.a. | no | | 0.070 | 1.35 |
| 37 | 209.1536 | n.a. | no | | 0.265 | 0.86 |
| 38 | 221.1532 | n.a. | no | | 0.183 | 1.45 |

| | | | | | |
|---|---|---|---|---|---|
| 39 | 221.19 | n.a. | no | 0.881 | 1.65 |
| 40 | 223.1327 | n.a. | no | 0.155 | 1.34 |
| 41 | 237.1123 | n.a. | no | 0.145 | 0.95 |
| 42 | 251.1641 | n.a. | no | 0.318 | 3.61 |

**Table S4:** Cohort characteristics with stratification criterion applied for the treated OSA group.

| | OSA untreated | OSA treated[a] |
|---|---|---|
| N | 43 | 29 |
| Age (y) | 61 (55.0, 69) | 65 (59, 70) |
| Sex, male, N (%) | 36 (84) | 25 (86) |
| BMI (kg/m$^2$) | 29.5 (27.0, 34.0) | 30.1 (27.8, 32.6) |
| AHI at diagnosis (events/h) | 30.2 (24.0, 45.0) | 35.0 (24.0, 13.5) |
| AHI at visit (events/h) | 28.0 (18.8, 40.0) | 1.2 (0.8, 4.4) |
| ESS at visit, points | 5.0 (3.0, 9.0) | 6.0 (2.5, 8.5) |

BMI: body mass index, AHI: apnea/hypopnea index, ESS: Epworth Sleepiness Scale. Values are presented as median ± interquartile range (IQR) unless otherwise stated. [a]stratification: averaged CPAP usage ≥ 5 h/night.

## Testing for normality and significant differences

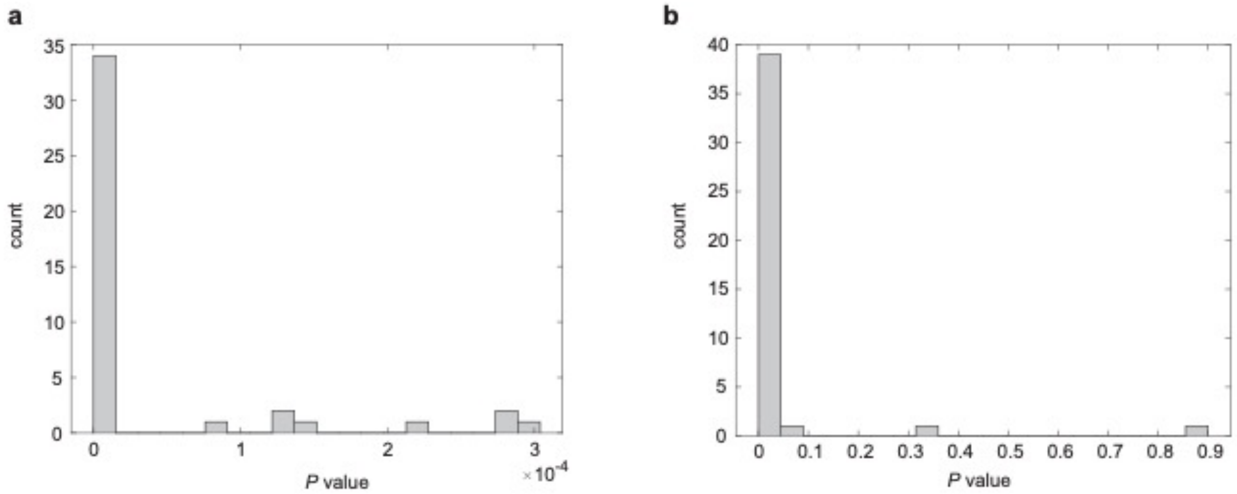


**Figure S1:** Shapiro-Wilk test for normality on the detected marker intensities in **a)** untreated and **b)** treated OSA. Stratification criterion: ≥ 5 h/night averaged CPAP usage.

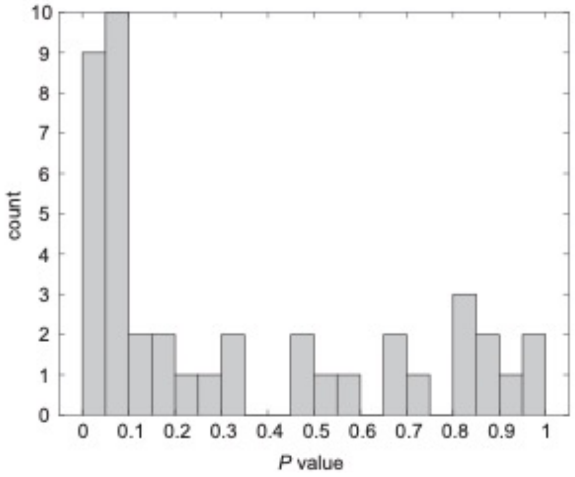


**Figure S2:** Mann-Whitney U test to determine significantly different OSA-associated metabolites between untreated and treated OSA patients. Stratification criterion: ≥ 5 h/night averaged CPAP usage, statistically significant markers: $P < 0.05$.

# Classification performance

1) Prediction for patients with treated and untreated OSA, unbalanced group size

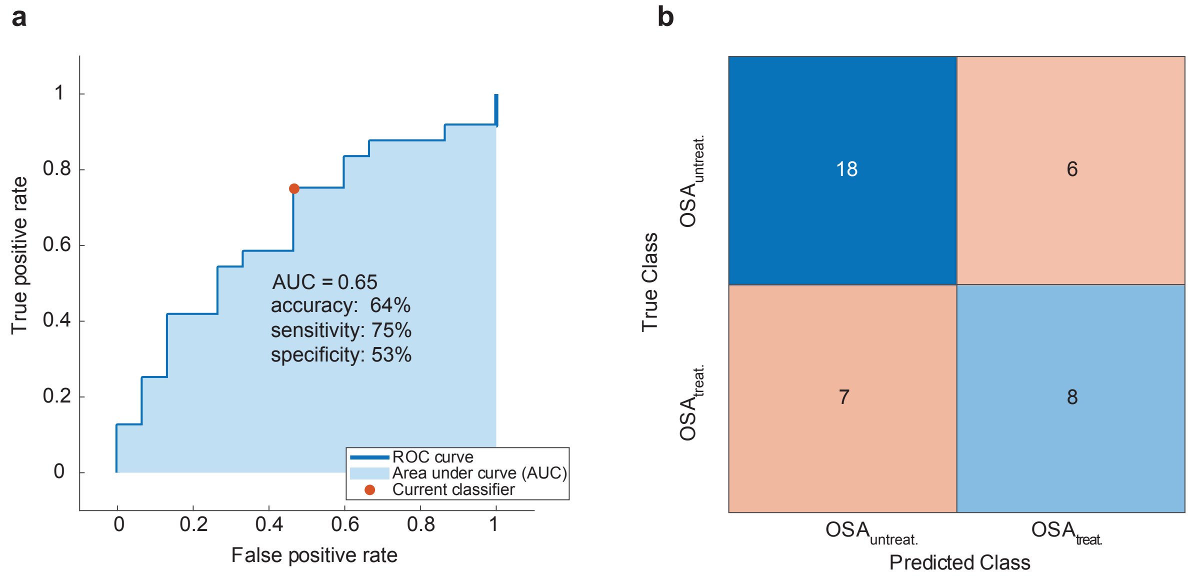


**Figure S3:** Classification performance of the 42 detected features for untreated OSA ($OSA_{untreat.}$, N=24) and treated OSA ($OSA_{treat.}$, N=15). Stratification criteria for the treated OSA group: ≥ 5 h/night averaged CPAP usage, and for both groups: AHI>30, or AHI>10 and ESS>10. **a)** Receiver operating characteristic (ROC) curve for OSA prediction from a 10-fold cross-validation applying the k-nearest neighbors algorithm resulted in an averaged area under the curve (AUC) of 0.65, **b)** confusion matrix for the prediction of untreated and treated OSA.

## 2) Prediction for patients with untreated OSA and control subjects

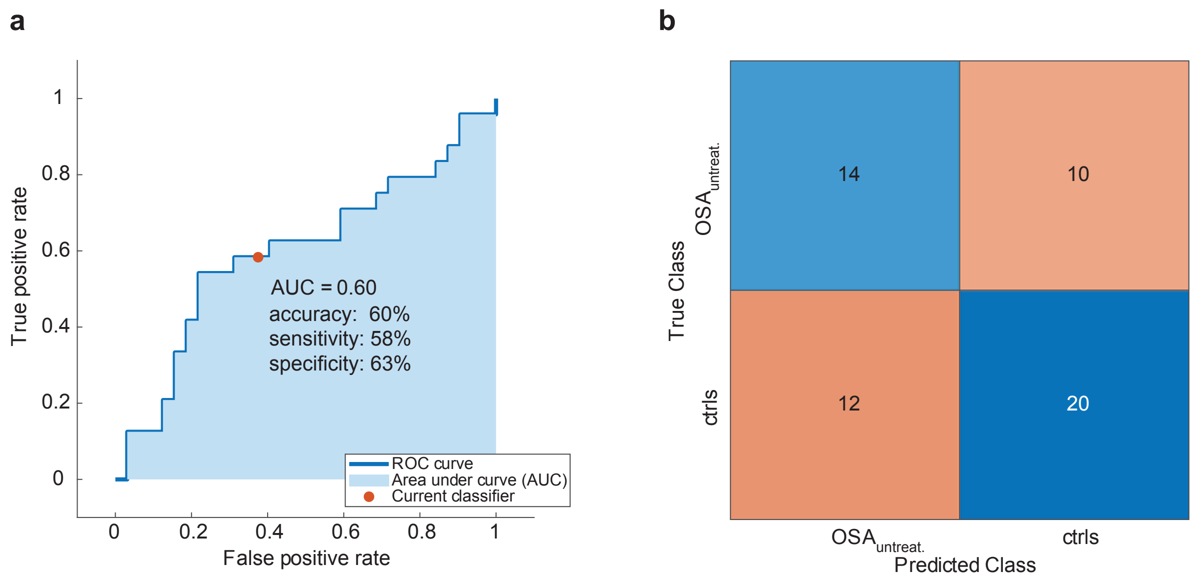


**Figure S4:** Classification performance of the 42 detected features for untreated OSA (N=24) and control subjects (ctrls, N=32). Stratification criteria for untreated OSA ($OSA_{untreat.}$): AHI>30, or AHI>10 and ESS>10. **a)** Receiver operating characteristic (ROC) curve for OSA prediction from a 10-fold cross-validation applying the ensemble methods resulted in an averaged area under the curve (AUC) of 0.60, **b)** confusion matrix for the prediction of untreated OSA and controls.

## 3) Predictions with different study groups

**Table S5:** Classification performances of 42 confirmed markers applied on the different study groups.

| | N (OSA) | N ($OSA_{treat.}$) | N (ctrls) | AUC | accuracy (%) | sensitivity (%) | specificity (%) |
|---|---|---|---|---|---|---|---|
| a) OSA, $OSA_{treat.}$ | | | | | | | |
| a.1) unbalanced | 24 | 15 | 0 | 0.65 | 64 | 75 | 53 |
| a.2) balanced | 15 | 15 | 0 | 0.80 | 77 | 73 | 80 |
| b) OSA, ctrls | | | | | | | |
| b.1) unbalanced | 24 | 0 | 32 | 0.60 | 60 | 58 | 63 |
| b.2) balanced | 24 | 0 | 24 | 0.61 | 60 | 63 | 58 |

Applied stratification criteria for untreated OSA (OSA) and treated OSA ($OSA_{treat.}$): AHI>30, or AHI>10 and ESS>10, additional for treated OSA: ≥ 5 h/night CPAP usage. AUC: area under the curve.
